# High-Resolution Echo-Planar Spectroscopic Imaging of the Human Calf

**DOI:** 10.1371/journal.pone.0087533

**Published:** 2014-01-30

**Authors:** Jan Weis, Morten Bruvold, Francisco Ortiz-Nieto, Håkan Ahlström

**Affiliations:** 1 Department of Radiology, Uppsala University Hospital, Uppsala, Sweden; 2 Philips Healthcare, Best, Netherlands; Glasgow University, United Kingdom

## Abstract

**Background:**

This study exploits the speed benefits of echo-planar spectroscopic imaging (EPSI) to acquire lipid spectra of skeletal muscle. The main purpose was to develop a high-resolution EPSI technique for clinical MR scanner, to visualise the bulk magnetic susceptibility (BMS) shifts of extra-myocellular lipid (EMCL) spectral lines, and to investigate the feasibility of this method for the assessment of intra-myocellular (IMCL) lipids.

**Methods:**

The study group consisted of six healthy volunteers. A two dimensional EPSI sequence with point-resolved spectroscopy (PRESS) spatial localization was implemented on a 3T clinical MR scanner. Measurements were performed by means of 64×64 spatial matrix and nominal voxel size 3×3×15 mm^3^. The total net measurement time was 3 min 12 sec for non-water-suppressed (1 acquisition) and 12 min 48 sec for water-suppressed scans (4 acquisitions).

**Results:**

Spectra of the human calf had a very good signal-to-noise ratio and linewidths sufficient to differentiate IMCL resonances from EMCL. The use of a large spatial matrix reduces inter-voxel signal contamination of the strong EMCL signals. Small voxels enabled visualisation of the methylene EMCL spectral line splitting and their BMS shifts up to 0.5 ppm relative to the correspondent IMCL line. The mean soleus muscle IMCL content of our six volunteers was 0.30±0.10 vol% (range 0.18–0.46) or 3.6±1.2 mmol/kg wet weight (range: 2.1–5.4).

**Conclusion:**

This study demonstrates that high-spatial resolution PRESS EPSI of the muscle lipids is feasible on standard clinical scanners.

## Introduction

Proton (^1^H) magnetic resonance spectra of lipids in skeletal muscle are dominated by two spectral lines originating from the methylene protons (-CH_2_-)n of extra- (EMCL) and intramyocellular (IMCL) lipids (EMCL_CH2_, IMCL_CH2_). Less intense resonances are also observable, originating from the methyl protons (CH_3_), methylene protons α and β to COO (-CH_2_-CH_2_-COO), α to C = C (-CH_2_-CH = CH-CH_2_-), diallylic methylene ( = CH-CH_2_-CH = ), and methine protons (-CH = CH-) [1], [2]. IMCL are stored in the form of small droplets in a magnetic susceptibility homogeneous environment (cytoplasm) of muscle cells. In such circumstances, the positions of IMCL spectral lines are independent in orientation of the myocytes relative to the external magnetic field B_0_ [2], [3]. The bulk fat (EMCL) is, in contrast, stored subcutaneously, in bone marrow and as interstitial (adipose) tissue. The anisotropic shape and geometrical arrangement of EMCL compartments result in a bulk magnetic susceptibility (BMS) shift of its spectral lines [3]. If the shape of the EMCL compartments resemble “infinitely long” cylinders, the spectral line shifts range from 0.2 ppm to −0.1 ppm between parallel and perpendicular orientations of the cylinders relative to B_0_ [3]–[6]. However, EMCL compartments in skeletal muscle do not follow a strict cylindrical shape. BMS effects could be therefore more complex. This expectation is supported by the study of Steidle et al [7]. The authors proposed a deconvolution method to compute magnetic field distribution (MFD) inside the voxel using the spectra of muscle lipids and bone marrow. Spectra of soleus muscle revealed that magnetic field deviations experienced by the EMCL compartments were in the interval from ca 0.6 to −0.2 ppm in respect of IMCL_CH2_. From Steidle et al study [7] it follows that EMCL spectral line shifts somewhat larger than 0.2 ppm or less than −0.1 ppm relative to IMCL can be expected.

The quantification of IMCL became important mainly because of its relation to insulin resistance in obese and non-obese adults as well as in non-diabetic offspring of type 2 diabetic subjects [8]–[10]. IMCLs are involved in lipid metabolism with a turnover of some hours. IMCL concentrations vary during physical exercise and also depend on training status, diet, fasting, oxygen supply, etc [4]. Fitting of IMCL resonances is often hampered by overlap with intense EMCL spectral lines. Single-voxel MRS with its large voxel size (>1 cm^3^) is the most sensitive to this effect [2], [6]. The voxel must be placed in the muscle regions free from large deposits of EMCL tissues. Improper voxel positioning will result in a spectrum with indistinguishable IMCL lines. To overcome this limitation, magnetic resonance spectroscopic imaging techniques (MRSI) were proposed [11]–[17]. The reduction of IMCL contamination was achieved by using smaller voxel sizes and by a post-acquisition selection of voxels with acceptable separation of IMCL_CH2_ line from EMCL_CH2_. Conventional STEAM, PRESS or slice selective 2D MRSI techniques reached nominal voxel sizes 5×5×10 mm^3^ using spatial matrices 32×32 or 36×36 [11]–[15]. Acquisition time was 20–35 minutes. The fundamental problem of MRSI is inter-voxel signal contamination (signal bleeding) of the strong EMCL signals from subcutaneous fat, bone marrow or larger facial layers into the adjacent regions of muscle due to limited k-space sampling. One solution to this problem is presaturation of large fat structures followed by removing the residual signals by a lipid extrapolation procedure [14], [15]. Another solution is to acquire MRSI data set with high spatial resolution [16], [17]. We previously used 2D MRSI based on a gradient echo sequence with step increments of TE [16]. Spectral matrix 128×256 led to the acquisition voxel size 1.2×0.6×15 mm^3^. Newcomer et al [17] used a conventional 2D MRSI sequence with decreased flip angles, short TR (146 ms), spectral matrix 64×64, and voxel size 2.5×2.5×10 mm^3^. The disadvantages of both high-resolution methods were poor water suppression, decreased spectral quality, and the inability to detect other than most intense EMCL_CH2_ and IMCL_CH2_ lines.

The drawbacks of conventional [11]–[15] and high-resolution MRSI techniques with short TR [16], [17] can be overcome by echo-planar spectroscopic imaging (EPSI) [18]–[23]. With the EPSI approach, a gradient echo train simultaneously encodes one spatial and one spectral dimension. It enables the acquisition of large spatial matrices with long TR in a short measurement time.

The focus of this study was to develop and implement a high-resolution 2D PRESS EPSI technique on a clinical MR scanner, to visualise the bulk magnetic susceptibility (BMS) shifts of EMCL spectral lines, and to investigate the feasibility of this method for the assessment of IMCL lipids.

## Methods

### Ethics Statement

The study was approved by The Regional Ethical Review Board in Uppsala and written consent was obtained from all participants.

### Subjects

Six male volunteers participated in this study. The subjects were healthy, non-obese, passive in free time physical activity and with sedentary occupations. Median age and body mass index were 30.5 years (range: 22–61), and 23.3±1.5 kg/m^2^ (range: 21–25.5), respectively.

### Data Acquisition

**Figure 1:**
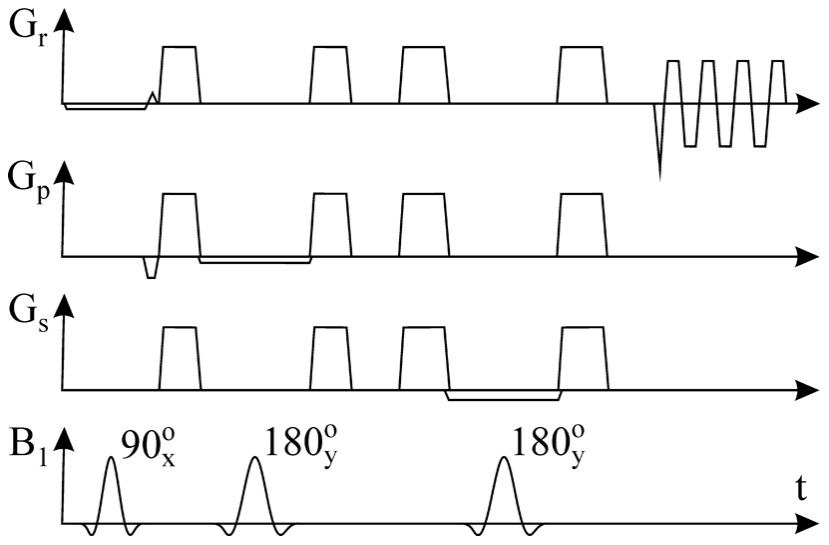
2D PRESS EPSI sequence. G_r_, G_p_, and G_s_ are gradients in read, phase-encoding, and slice selection directions, respectively. B_1_ depicts excitation rf pulses.

**Figure 2:**
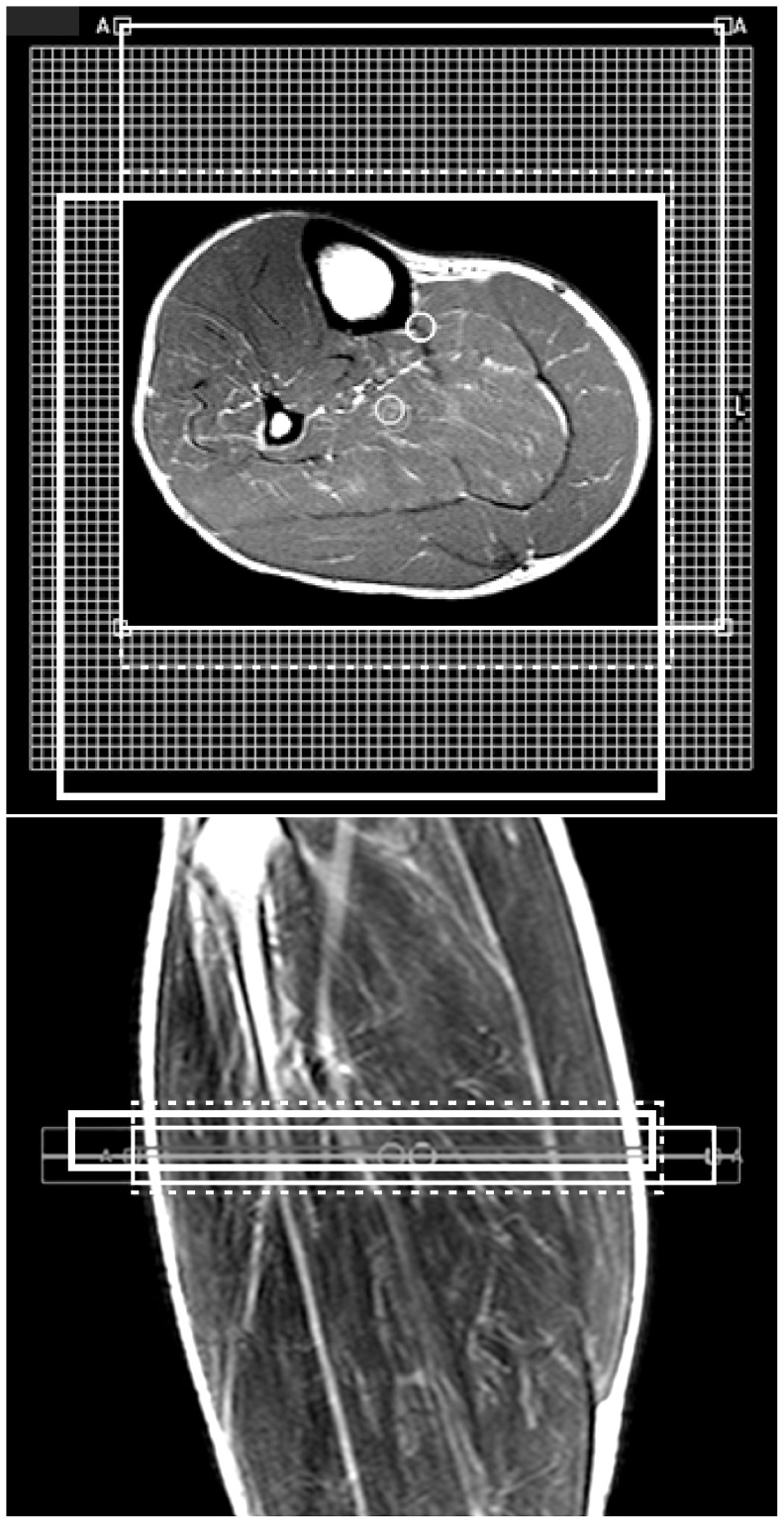
Planning of 2D PRESS EPSI experiment with a spatial matrix 64×64 on the background of T_1_-weighted transversal and coronal spin echo images. Large thin and thick rectangles show lipid (-CH_2_-)n and TMA PRESS boxes, respectively. Dashed rectangle depicts shimming volume.

All the experiments were performed by using a 3 T scanner (Achieva, Philips Healthcare, Best, Netherlands). The trapezoidal echo-planar readout gradient train was implemented into a manufacturer point-resolved spectroscopy (PRESS) localization module (Fig. 1). It enabled utilization all possibilities of the manufacturer spectroscopic imaging package including water/fat and outer volume suppression modules, excitation pulse shapes, etc. The PRESS localization technique was chosen because this method effectively reduces risk of signal contamination from outside the VOI, e.g. from the other leg (in our case). However, PRESS excitation (Fig. 1) was effective only for the slice selection (Fig. 2), because our intention was to perform spectroscopic imaging in the whole slice (calf). A whole-body coil was used for excitation, and the standard, manufacturer provided flexible two-element coil (20 cm diameter) placed in front of and behind the calf served as the receiver. The subject lay in a supine position with the most extended part of the calf in the center of the receiver coil. 2D PRESS EPSI measurement was performed in the transversal plane by means of 64×64 spatial matrix (Fig. 2); FOV 192 mm; nominal voxel size 3×3×15 mm^3^ (0.135 cm^3^); acquisition bandwidth 128 kHz; TR 1500 ms, and TE 38 ms (minimum TE). One and four signal averages were used for non-water and water suppressed scans, respectively. Typical size of the excitation volume (PRESS box) was 160×160 mm in the transversal plane (Fig. 2). Thickness of the PRESS box was 15 mm in feet-head direction. Chemical shift displacement between PRESS box of trimethyl ammonium containing compounds (TMA) at ca 3.2 ppm and methylene (-CH_2_-)n PRESS box (∼1.5 ppm) was 44.9, 16.4, and 4.2 mm in anterior-posterior, left-right, and feet-head directions, respectively (Fig. 2). 90^o^ RF-pulse with broader bandwidth (BW) produces lower chemical shift artefact in the left-right direction than 180^o^ RF-pulse with narrow BW in the anterior-posterior direction (Fig. 2). Radiofrequency of the PRESS pulses was centered to ∼1.5 ppm in water-suppressed scans, i.e. there was no chemical shift displacement between the water and methylene (-CH_2_-)n PRESS boxes. The period of readout gradients was 1.6108 ms and readout gradient train consisted of 128 positive and 128 negative lobes. Trapezoidal readout gradient waveforms consisted of linear ramps of 0.1566 ms duration, a constant plateau of 0.4922 ms duration and an amplitude 15.66 mT/m. The duration of the linear ramps was limited by the slew rate of 100 mT/m/s fixed by manufacturer. Conventional phase-encoding was performed in the anterior-posterior direction (Fig. 1). Magnetic field homogeneity in the whole slice was improved by iterative first order shimming (Fig. 2). Typical linewidth of the water line was between ∼15 and ∼25 Hz. Water suppression was performed by applying two bandwidth selective RF pulses and spoiler gradients. Suppression was achieved by adjusting the tip angle of the second RF pulse such that the longitudinal magnetization of the water signal was minimal at the time of the first PRESS excitation RF pulse. Water suppression bandwidth was 140 Hz. An acquisition of two interleaved gradient echo trains was performed in order to increase spectral bandwidth [21]. The beginning of the second echo train was shifted by a half period (0.8054 ms) of the readout gradients. Combination of both echo trains resulted in the effective inter-echo time 0.8054 ms corresponding to a spectral bandwidth of 9.72 ppm. The total net measurement time of our sequence with two interleaves of gradient echo train was 3 min 12 sec for non-water-suppressed (1 acquisition) and 12 min 48 sec for water-suppressed scans (4 acquisitions).

### Data Processing

**Figure 3:**
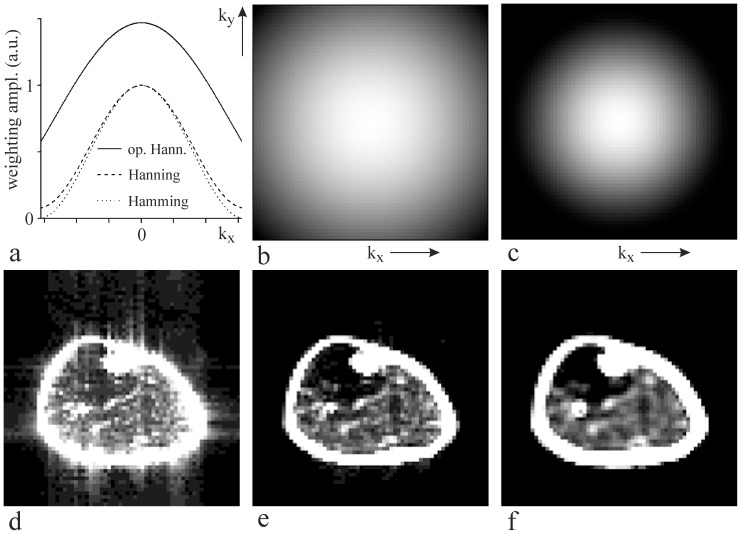
K-space filters of raw data and correspondent fat images. (a) Profile of the usual k-space weighting functions: optimized Hanning (full line), standard Hanning (dashed line) and Hamming (dotted line). (b) Optimized 2D Hanning filter. (c) Standard 2D Hanning filter. (d) Fat image computed without application of k-space filter. (e) Fat image computed from filtered data set using an optimized 2D Hanning filter. (f) Fat image computed from filtered data set using a standard 2D Hanning filter.

**Figure 4:**
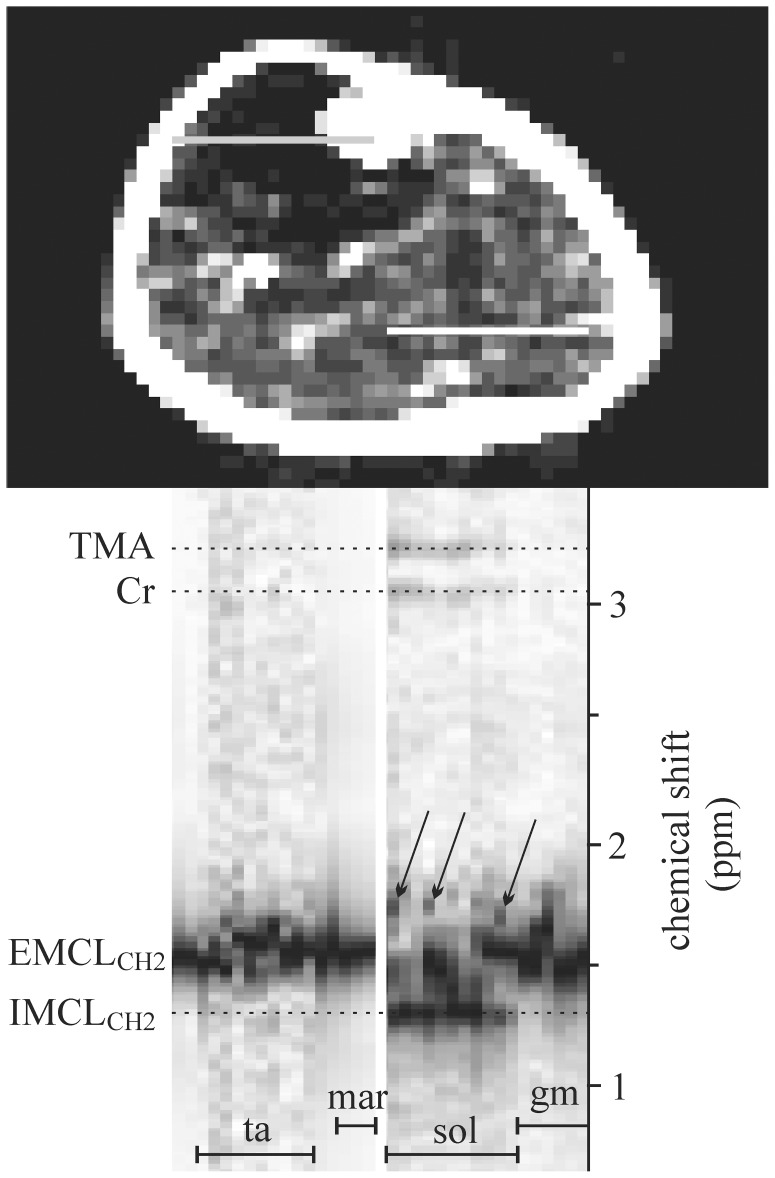
Magnitude spectra (vertical columns) of the voxel rows, depicted by gray and white line in the corresponding fat image. Spectra represent, tibialis anterior (ta) muscle, bone marrow (mar), soleus (sol) and gastrocnemius medialis (gm) muscles. Spectra were corrected for shifts caused by magnetic field deviations. EMCL_CH2_ sub-peaks in soleus can be seen between 0.4 and 0.5 ppm, from IMCL_CH2_ line (arrows).

Measured raw data were transferred to a Linux workstation for processing. Data processing software was developed in-house. Measured data were reorganized to the data sets containing either odd or even echoes. The first 128 echoes arising either from positive or negative readout gradients were processed for each element of the receiver coil separately, i.e., four (k_read_, k_phase_, k_t_) = (64, 64, 128) data matrices were processed. Measured matrices were zero-filled to size (64, 64, 512). Data processing began with optimized 2D Hanning filter [24] applied across the k_read_ and k_phase_ directions to reduce signal bleeding. Radial profile (Fig. 3a) of the filter (Fig. 3b) was computed using the weighting function [24]: w(k_n_) = β/2[1+ cos(2πk_n_Δx/α)], where k_n_ = [n – (N +1)/2]Δk; n = 1, 2, …, N; N is the number of phase-encoding steps; Δk = 1/FOV is the k-space increment; Δx = FOV/N is the nominal spatial resolution, α = 1.71, and β = 1.47. The first FFT was performed along the k_t_ axis. The chemical shift artifacts caused by readout gradients were removed using a first-order phase correction [25], [26]. Data processing continued with 2D FFT along k_read_ and k_phase_ dimensions. The spatial distribution of the magnetic field ΔB(x, y) in the measured slice was computed from the position of the highest spectral line of non-water suppressed magnitude spectra [25], [26]. The voxel spectra were then corrected by shifting about ΔB/B_0_ along the spectral axis (Fig. 4). The magnitude spectra of the resulting four spectral matrices were then averaged. Averaging of real spectra was avoided because large quantities of voxel spectra (4×64×64) cannot be reliably phase-corrected before summation. Eddy currents corrections were unnecessary. Unsuppressed water line (not shown) as well as the most intense lipid lines did not reveal signs of eddy current distortions.

### Spectrum Processing

**Figure 5:**
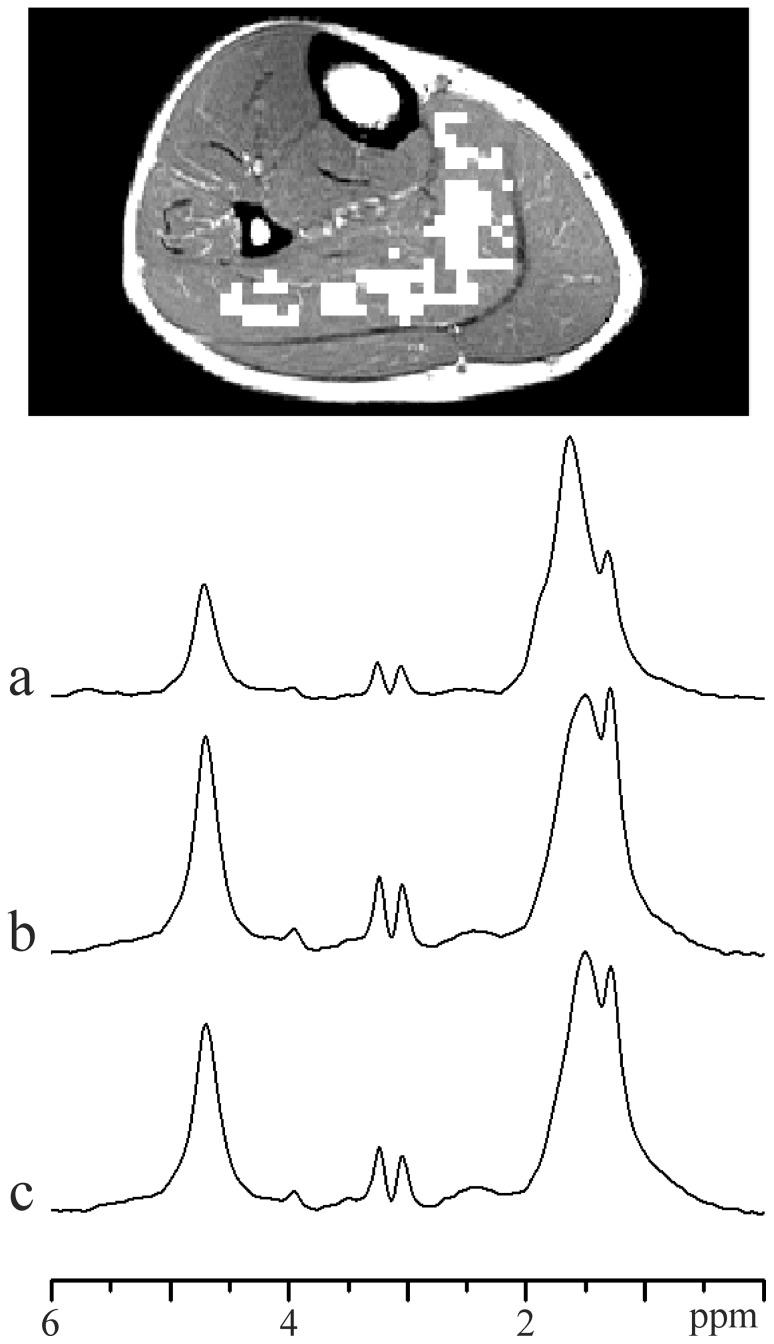
Magnitude spectra of soleus muscle (108 voxels). VOI indicate white MRSI voxels in T_1_-weighted image. (a) Spectrum computed without application of k-space filter during data processing. (b) Spectrum computed from filtered data set, using an optimized 2D Hanning filter. (c) Spectrum computed from filtered data set, using a standard 2D Hanning filter.

**Figure 6:**
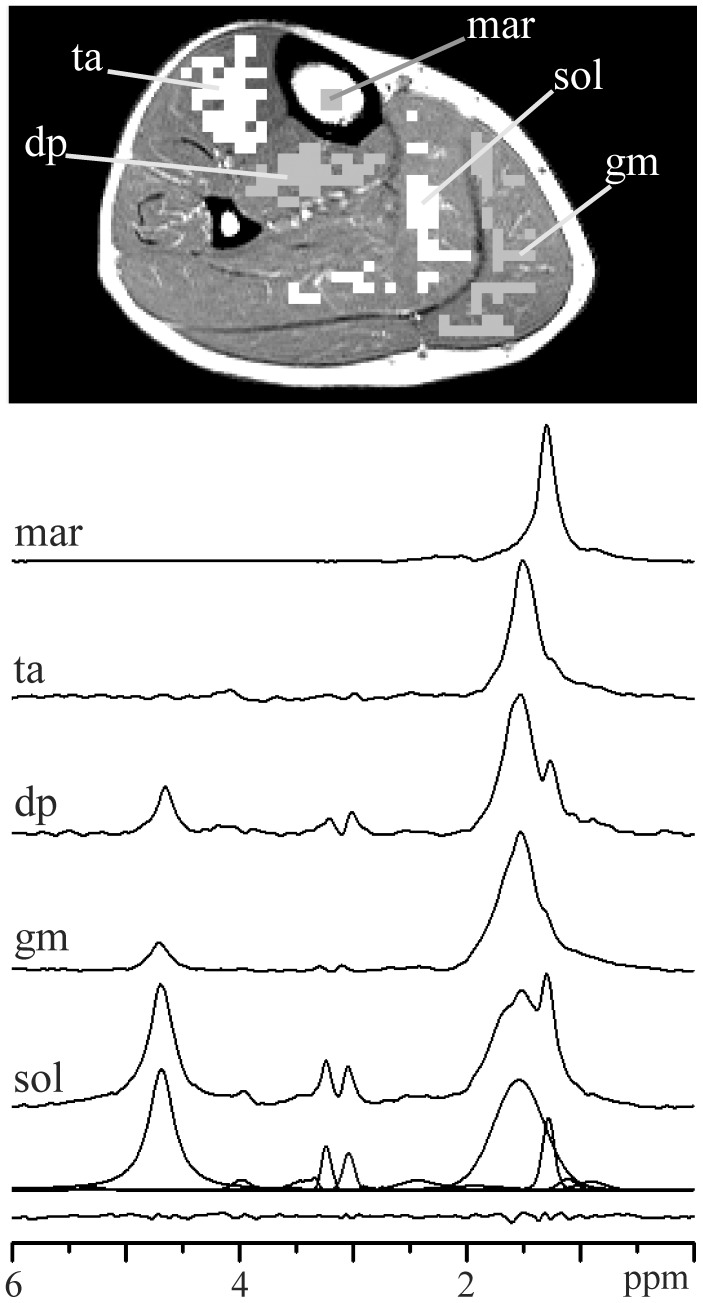
Magnitude spectra of bone marrow (mar, 4 voxels) and in tibialis anterior (ta, 45 voxels), deep posterior compartment (dp, 36 voxels), gastrocnemius medialis (gm, 40 voxels), and soleus (sol, 42 voxels) muscles. Spectrum of soleus muscle is completed by LCModel fits and residue. VOIs indicate white and gray MRSI voxels in T_1_-weighted image.

**Figure 7:**
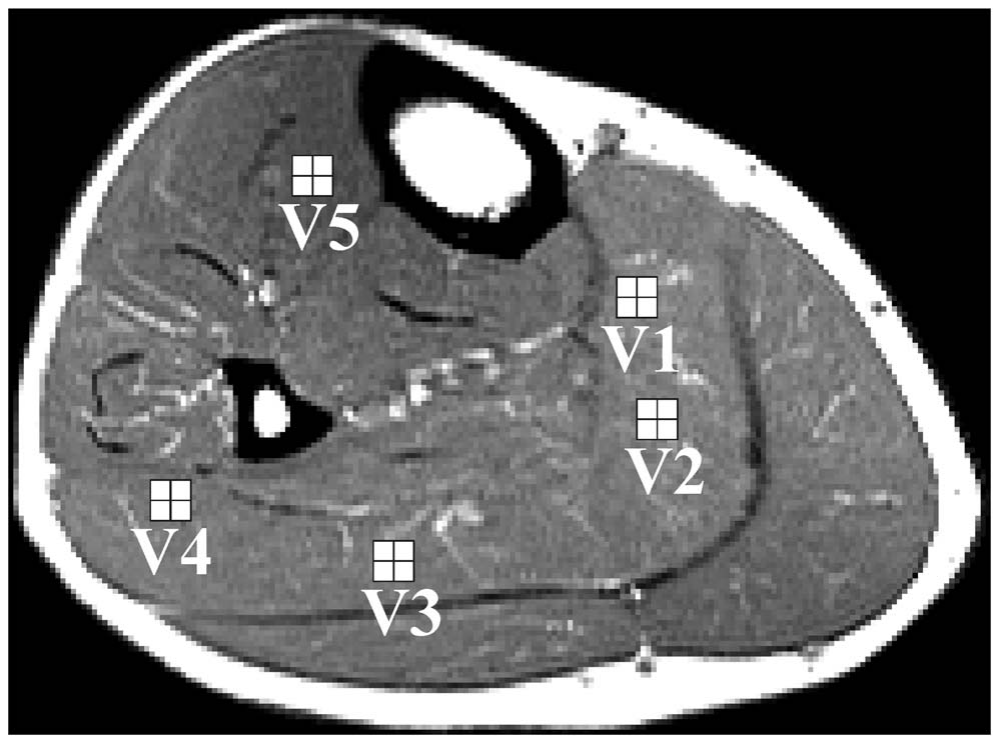
Volumes of interest employed for computing the spectra shown in figures 8 and 9. VOIs V1–V5 contains four MRSI voxels (white squares).

Fat images (Fig. 3) were computed by integrating EMCL_CH2_ and IMCL_CH2_ spectral lines. Irregularly shaped VOIs were drawn in the fat image (Fig. 3e) after appropriate thresholding of the fat intensities, i.e. the highest fat intensities were removed. Positions of the VOIs were then visualized by superposition with corresponding T1W images. Figures 5–7 show VOIs used in this study. The average magnitude spectra per voxel were computed by summation of the voxel spectra from the defined volume of interest (VOI) and division by the number of voxels. The spectra of soleus muscle were chosen for quantification. The threshold levels applied for soleus VOI selections were accepted when the EMCL_CH2_ spectral line amplitude was comparable to the IMCL_CH2_ (Fig. 5b, c). Spectra were fitted by LCModel [27] in the interval <−0.2; 8>ppm. No apodization of the FIDs to improve SNR was used in this study. Complex FIDs for LCModel were computed by inverse FFT of the magnitude spectra [16]. The fat content in volume percentage (vol%) was computed using the EMCL_CH2_ intensity of the voxels with a 100% fat content as an internal reference [16]. The reference fat spectrum was obtained from the voxel group indicated by grey pixels in the central part of tibial bone marrow (Fig. 6). The bone marrow fat content of 1.17 mol/kg wet weight was used to convert vol% into mmol/kg wet weight [15].

## Results

The proposed 2D PRESS EPSI technique and data processing provided spectroscopic fat images (Fig. 3e) with acceptable spatial resolution and magnitude spectra with a very good signal-to-noise ratio (SNR) in all subjects. Figure 3a shows the profile of the usual 2D k-space weighting functions: standard Hanning, Hamming and optimized Hanning [24]. Optimized and standard 2D Hanning filters are shown in Fig. 3b and c, respectively. Figure 3d represents the fat image computed from the data set without application of k-space filter. From Fig. 3d it follows that the signal bleeding is a non-negligible problem although k-space sampling was increased to 64×64. Corresponding fat images of filtered data sets, using an optimized and standard 2D Hanning filters, are shown in Fig. 3e and f, respectively. Figures 3e, f demonstrate that k-filters reduce inter-voxel signal contamination, but decrease spatial resolution. In this study, a 2D optimized Hanning filter (Fig. 3b) was applied as a best compromise between signal bleeding suppression, spectrum quality and spatial resolution [24]. This filter increased the nominal voxel size by a factor 1.71 in both spatial directions [24]. Our “true” voxel shape could be best approximated as a cylinder with a diameter of 5.13 mm and height 15 mm (volume 0.31 cm^3^). From comparison of Fig. 3e and f, it is evident that standard Hanning filter produces significantly larger true voxel size than optimized Hanning. The representative spectra of a normal volunteer are shown in Fig. 5 and 6. The most pronounced lines are residual water (4.7 ppm), singlet of creatine methylene protons (3.9 ppm), TMA (3.2 ppm), total creatine (tCr) (3 ppm), broader EMCL_CH2_ line at ∼1.5 ppm, and IMCL_CH2_ at 1.3 ppm.

Figure 5 demonstrates the impact of k-space filters on the spectrum of soleus muscle. The spectrum computed without application of k-space filter (Fig. 5a) reveals increased EMCL_CH2_ line at ∼1.5 ppm and a little shoulder at ∼1.8 ppm. This is due to the signal bleeding from the neighboring voxels to the selected VOI. Signal bleeding was suppressed by optimized (Fig. 5b), as well as by standard Hanning filter (Fig. 5c), as follows from missing shoulder at ∼1.8 ppm and reduced EMCL_CH2_ line. From a comparison of Fig. 5b and c, it follows that optimized Hanning filter (Fig. 3b) sufficiently suppressed inter-voxel signal contamination because significant sharpening of k-space filter, by standard Hanning (Fig. 3c), did not lead to a further decrease in the EMCL_CH2_ line. In fact, EMCL_CH2_ intensity increased slightly (Fig. 5c) due to larger true voxel (VOI) size.

Figure 6 shows the reference fat spectrum computed from the central tibial bone marrow and muscles in the tibialis anterior, deep posterior compartment, gastrocnemius medialis, and soleus. The spectrum of the soleus muscle is completed by LCModel fits and residue. The mean soleus IMCL content of our six volunteers was 0.30±0.10 vol% (range: 0.18–0.46) or 3.6±1.2 mmol/kg wet weight (range: 2.1–5.4). The Cramér-Rao lower bounds (CRLB) of the LCModel fits of all evaluated soleus spectra were ≤2% and ≤7% for EMCL_CH2_ and IMCL_CH2_ lines, respectively. Slight different IMCL concentration 0.26±0.1 vol% (range: 0.17–0.42) reported previously [28] was caused by different chemical shift interval <0.2; 4>ppm used in LCModel data processing.

**Figure 8:**
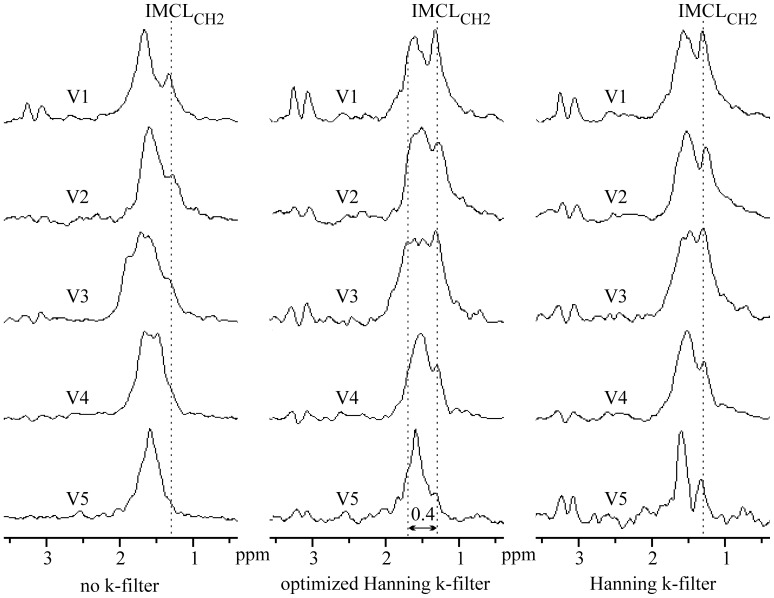
Magnitude spectra computed from the VOIs V1–V5 (four voxels). Left panel represents spectra computed without application of k-space filter during data processing. Middle and right panel depict spectra computed from filtered data set using an optimized and standard 2D Hanning k-space filter, respectively. Figure 6 shows VOIs.

**Figure 9:**
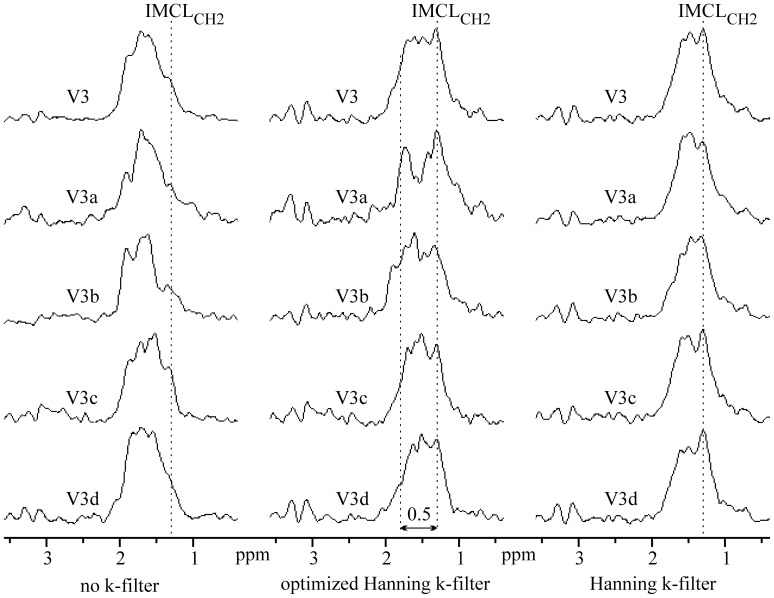
Magnitude spectra computed from the volume of interest V3 (four white voxels in Fig. 7) and its individual voxel spectra V3a (left up), V3b (right up), V3c (left bottom), and V3d (right bottom). Left panel represents spectra computed without application of k-space filter during data processing. Middle and right panel depict spectra computed from filtered data set using an optimized and standard 2D Hanning k-space filter, respectively.

The volumes of interest V1–V5 (Fig. 7) were chosen in the regions with more complex BMS shifts of EMCL_CH2_ spectral lines. The purpose of such VOI selections was to find spectra with split EMCL_CH2_ lines and with increased BMS shifts. The left panel in Fig. 8 contains spectra computed without application of k-space filter during data processing. Middle and right panel show spectra computed from filtered data set using an optimized and standard 2D Hanning k-space filter, respectively. Figure 9 shows spectra from the VOI V3 together with their individual single-voxel spectra V3a-V3d. Whereas the left panel depicts spectra computed without k-filtering of raw data, middle and right panel contains spectra obtained from the filtered data set using an optimized and standard 2D Hanning k-filter, respectively. Impact of k-filter is in figures 8 and 9 visualized by significant spectral shape differences between “raw” spectra in the left panels and “k-filtered” spectra in middle and right panels. Differences between shapes of the spectra in middle and right panel can be explained by a larger true voxel size of the spectra computed from data sets filtered by a standard 2D Hanning k-filter compare to optimized Hanning filter. The spectra in Fig. 8 and 9 reveal splitting of the EMCL_CH2_ lines and their position shifts up to 0.4–0.5 ppm from the IMCL_CH2_ line. Such spectra were found in all volunteers.

## Discussion

Slice selective EPSI of the human brain was performed in the majority of previous studies [20], [22], [23], [29]–[32]. Maximum size of spatial matrices was 32×32 and 32×32×16 in 2D and 3D experiments, respectively. Subcutaneous fat suppression was achieved by outer volume suppression (OVS) slices and/or by a broadband inversion pulse. Acquisition times varied between ∼8 and ∼30 minutes. With the recent improvements in gradient hardware performance, it was demonstrated, that spectral quality (linewidths) of brain spectra and SNR per unit time and unit volume were similar to those obtained with conventional phase-encoded PRESS MRSI techniques [23]. Low spectral intensities of brain metabolites hindered utilizing the speed of EPSI techniques. It should be noted, that readout bandwidth (echo sampling frequency), instead of spectral bandwidth, is the determining factor for SNR of EPSI techniques [33]. This leads to unavoidable loss in SNR compared to conventional MRSI with pure phase-encoding of signals and readout in the absence of imaging gradients.

EPSI techniques are, however, useful in the applications where the penalty paid in SNR loss is an acceptable trade for the considerable acquisition time reduction. Such applications are spectroscopy of lipids in skeletal muscle, liver, heart, etc. The net measurement time of our 2D PRESS EPSI sequence with two interleaves of gradient echo train was 12 min 48 sec for water-suppressed scans (4 acquisitions). However, the spectra of the bone marrow and muscles in Fig. 6 reveal SNR 114, and ≥28, respectively. SNR was computed by LCModel. The measurement time of water-suppressed scans could therefore be reduced by factor 2 (2 acquisitions). For comparison, minimum measurement time needed for conventional 2D MRSI (TR 1500 ms, spectral matrix 64×64, 1 acquisition) is 102 min 24 sec for non-water-suppressed and the same time for water-suppressed scans.

Our module spectra have sufficient spectral resolution to distinguish TMA from tCr, IMCL_CH2_ from EMCL_CH2_, and to detect EMCL_CH2_ splitting. TMA and tCr spectral intensities reveal differences between muscles (Fig. 6, 8, 9) and dipolar coupling effects, in agreement with previous studies [34], [35]. Both spectral lines are highest in soleus muscle and lowest in gastrocnemius medialis and tibialis anterior. Sensitivity of our EPSI sequence was not sufficient to detect TMA and tCr lines in all muscles due to small voxel size and a short acquisition time. TMA and tCr intensities were at the level of the noise in gastrocnemius medialis and tibialis anterior muscles, as follows from the individual voxel spectra shown in Fig. 4. Summation of such voxel spectra cannot improve the detectability of TMA and tCr.

Broad EMCL_CH2_ and narrow IMCL_CH2_ lines dominate in typical spectra of m. soleus (Fig. 5, 6). EMCL_CH2_ originates from extracellular fatty strands located between muscle fibers. It was suggested that these strands can be modeled by “infinite long” cylinders with the long axis at an angle θ relative to the direction of the static magnetic field B_0_ [5], [6]. According to this model, the EMCL_CH2_ shift ranges from 0.2 ppm to −0.1 ppm relative to IMCL_CH2_ corresponding to a change in orientation 0^o^ ≤ θ ≤90^o^ [5], [6]. EMCL_CH2_ position and linewidth are determined by the dominant angle θ and by a degree of dispersion in orientations of fatty strands within the volume of interest, respectively [36]. The model of long cylinders agrees well with the spectra obtained from the large volumes of interest (Fig. 5, 6). However, this model is unable to explain spectra originating from small VOIs shown in Fig. 8 and 9. EMCL_CH2_ signals are split into more well resolved peaks. Moreover, EMCL_CH2_ shifts are increased up to 0.4–0.5 ppm relative to IMCL_CH2_. These results are in agreement with the theoretical simulation and experimental evidence published by Steidle et al [7]. The authors found that EMCL compartments in soleus muscle experienced magnetic field deviations in the interval from ca 0.6 to −0.2 ppm in respect of IMCL_CH2_. It should be also noted that EMCL_CH2_ spectral line splitting and shifts up to ∼0.3 ppm relative to IMCL_CH2_ can be seen in the spectra of Hwang et al [12]. However, the authors did not point out or discuss these spectral features.

It should be emphasized that EMCL_CH2_ spectral line splits and their shifts up to 0.4–0.5 ppm relative to IMCL_CH2_ cannot be explained by signal bleeding from regions with strong lipid signals, in combination with B_0_ shifts in the voxels. High effectiveness of signal bleeding suppression using Hanning filters was demonstrated theoretically as well as experimentally in the previous studies [24], [37]. In addition, the significance of signal bleeding artefacts decreases with increasing size of acquisition matrix. In our case was signal bleeding negligible due to combination of large acquisition matrix 64×64 and application of the Hanning k-filter. Figures 3e, f, and spectra in Fig. 4, 5, 8, 9 demonstrate high effectiveness of the Hanning k-filters in signal bleeding suppression. Figure 4 shows BMS shifts of the EMCL_CH2_ lines in more detail. This phenomenon is best seen in the voxel spectra of the soleus muscle. While TMA, tCr, and IMCL_CH2_ intensities are in the correct positions (in line), EMCL_CH2_ peaks at the same voxels show splitting, dispersion and shifts in vertical (ppm) direction. Some EMCL_CH2_ sub-peaks can be seen between 0.4 and 0.5 ppm from the correspondent IMCL_CH2_ line (arrows in Fig. 4). This finding reveals that BMS effects are more complex than previously demonstrated. Spectra measured from the large VOIs (>1 cm^3^) reveal EMCL_CH2_ shifts up to 0.2 ppm. In such large volumes dominates long plate EMCL structures which resemble “infinite long” cylinders. However, very small and isolated EMCL clusters could exist in the lean muscle. These spatial structures do not resemble infinite long cylinders. We suppose that another kind of BMS effects could occur on the discontinuities or interfaces of such fat clusters.

Quantification of IMCL content was restricted to m. soleus because this muscle group was studied in many previous studies, and because of its center position between the flexible receiver coils, i.e. in the most homogeneous region of the receiver coils sensitivity. Mean IMCL concentration of our six normal volunteers was, 0.30±0.10 vol% or 3.6±1.2 mmol/kg, wet weight. This result agrees well with published values [12], [15], [16]. Despite good agreement, our result has to be taken with some caution because spectral intensities were not corrected for spatial sensitivity of the receiver coils. Measurements of coils sensitivities are challenging. In our case, this is because the mutual position of the flexible coils is different in each measurement session and the water/fat intensity ratio is different in each voxel. This problem could be circumvented by using a quadrature receiver knee coil. Such coil was not available in our laboratory at the time of this study.

To our knowledge, this is the first application of the EPSI technique in quantification of muscle lipids. PRESS localization was used only for slice selection. We were not forced to select PRESS box inside the slice because of negligible signal bleeding from subcutaneous fat and bone marrow. It enabled selection the bone marrow spectrum as the internal concentration reference. Combination of EPSI and PRESS VOI selection is relatively unusual. Only three research groups used the PRESS EPSI approach previously [38]–[41]. PRESS excitation improves VOI definition, reduces risk of signal contamination from outside the VOI, enables omitting inversion fat suppression pre-pulse and reduces the number of OVS slabs or even avoids them in spectroscopy of the brain [39], [41], prostate [40], heart [42], etc. More complicated data processing of EPSI techniques is in our application counterbalanced by high spatial resolution and by significantly shorter measurement time compared to conventional MRSI. The high spatial resolution enables noncontiguous and irregularly shaped VOIs (Fig. 5, 6). It was possible to improve the reliability of the muscle lipid estimations by avoiding the influence of relaxation effects because the fat (bone marrow) spectral line was used as the internal concentration reference.

## Conclusion

The EPSI sequence with PRESS volume of interest localization was developed and implemented in a 3T scanner. The use of a large spectral matrix 64×64, together with the optimized Hanning k-filter significantly reduced inter-voxel signal contamination of the strong EMCL signals from subcutaneous fat, bone marrow or layers of adipose tissues. The small voxels enabled visualisation of EMCL methylene (-CH_2_-)n spectral lines splitting and their BMS shifts up to 0.5 ppm relative to correspondent IMCL methylene line. This study demonstrates that high-spatial resolution PRESS EPSI of the muscle lipids is feasible on clinical scanners.
